# Asiatic acid protects oocytes against *in vitro* aging-induced deterioration and improves subsequent embryonic development in pigs

**DOI:** 10.18632/aging.202184

**Published:** 2020-12-03

**Authors:** Wei-Yi Hu, Xiao Xia Li, Yun Fei Diao, Jia-Jia Qi, Da-Li Wang, Jia-Bao Zhang, Bo-Xing Sun, Shuang Liang

**Affiliations:** 1 Department of Animals Sciences, College of Animal Sciences, Jilin University, Changchun, China; 2 College of Animal Science and Technology, Jilin Agriculture Science and Technology University, Changchun, China; 3 Jilin Province Key Laboratory of Preventive Veterinary Medicine, Jilin Agriculture Science and Technology University, Changchun, China

**Keywords:** asiatic acid, oocyte aging, oxidative stress, pig, early embryonic development

## Abstract

As a pentacyclic triterpene in *Centella asiatica*, asiatic acid (AA) is a powerful antioxidant with many bioactivities. In the present research, we investigated whether AA has the potential to rescue the decrease in porcine oocyte quality that occurs during *in vitro* aging (IVA). Mature porcine oocytes were collected and then continuously cultured for an additional 24 h or 48 h with or without AA in maturation medium as an IVA model. The results revealed that AA supplementation reduced the percentage of abnormal aged porcine oocytes during IVA. Furthermore, AA supplementation effectively maintained aged porcine oocyte developmental competence, both parthenogenetic activation and *in vitro* fertilization. The number of sperm that bound to the zona pellucida on aged porcine oocytes was higher in the AA-supplemented group than in the non-supplemented group. Moreover, AA supplementation not only blocked IVA-induced oxidative stress but also maintained intracellular GSH levels and reduced the percentage of early apoptosis aged porcine oocytes. Mitochondrial functions were disordered during the IVA process. The intracellular ATP levels and mitochondrial membrane potential in aged porcine oocytes were dramatically increased by AA supplementation. Therefore, AA has beneficial effects on porcine oocyte quality and developmental potential maintenance during IVA.

## INTRODUCTION

Currently, the use of assisted reproductive technology (ART) is widespread [1–3]. It is inevitable for oocytes used in ART to undergo *in vitro* culture before fertilization. If oocytes are not fertilized at the optimal period, unfertilized oocytes show a time-dependent decline in quality; this phenomenon is called *in vitro* oocyte aging [4, 5]. Oocyte quality determines the developmental capacity of the embryo after fertilization [4–6]. Compared with high-quality oocytes, poor-quality oocytes have reduced antioxidation activity and weak developmental competence [7, 8].

Many studies point to a series of changes in aged oocytes compared with fresh oocytes, such as zona pellucida hardening [9], mitochondrial dysfunction [6, 10], spindle abnormalities [5, 11] and chromosome aneuploidy [12]. Recent studies have shown that a decline in antioxidant capacity occurs during the oocyte aging process [13], which produces superfluous free radicals, including reactive oxygen species (ROS), inducing many cascades that influence oocyte quality, such as DNA damage, and apoptotic pathways [14–16]. Oocyte mitochondria are very vulnerable to oxidative stress and DNA damage, and dysfunctional mitochondria impair the production of adenosine triphosphate (ATP); moreover, an insufficient ATP supply is an important reason for decreased oocyte fertilization competence and altered embryo development [17]. Oxidative stress is also a factor that induces apoptosis and chromosome instability [18]. It is well known that antioxidants can partially counteract the negative effects of oxidative stress [19, 20]. Therefore, the balance between antioxidant and pro-oxidant substances is a critical factor influencing the quality of aged oocytes.

As a pentacyclic triterpene in *Centella asiatica* [21], asiatic acid (AA) has been shown to have many bioactivities, including antiapoptotic [22], anticancer [23], anti-inflammatory [24], neuroprotective [25], antifibrotic [26], and hypoglycemic action [27, 28]. In addition, AA effectively protects against oxidation [29], which could guard against the damage to the neuronal system resulting from quinolinic acid [30], alleviate seizures caused by kainic acid [31], and play an important role in preventing LPS/d-GalN-induced fulminant hepatic failure (FHF) by limiting oxidative stress [32]. Furthermore, our previous results suggested that AA exerts beneficial effects on porcine early embryonic development by ameliorating oxidative stress and preserving mitochondrial function [33]. It has been reported that AA might also be beneficial to the prevention or alleviation of brain aging [34].

Despite the well-known biological characteristics and physiological functions of AA and its beneficial protective effects, the protective effect and underlying mechanisms of AA on mammalian oocytes have not been defined. Thus, we hypothesized that AA could protect porcine oocytes from deterioration during the *in vitro* aging (IVA) process and could improve subsequent embryonic developmental competence. In the present research, we first explored the effects of AA supplementation during the IVA process on morphological changes and subsequent developmental competence of aged porcine oocytes. Second, we further analyzed the effects of AA on oxidative stress, apoptosis and mitochondrial function in porcine oocytes via the IVA model to identify the associated underlying mechanism. The results could shed light on the underlying beneficial effects of AA on aged oocyte quality.

## RESULTS

### AA reduces morphological defects during the IVA process of porcine oocytes

**Figure 1:**
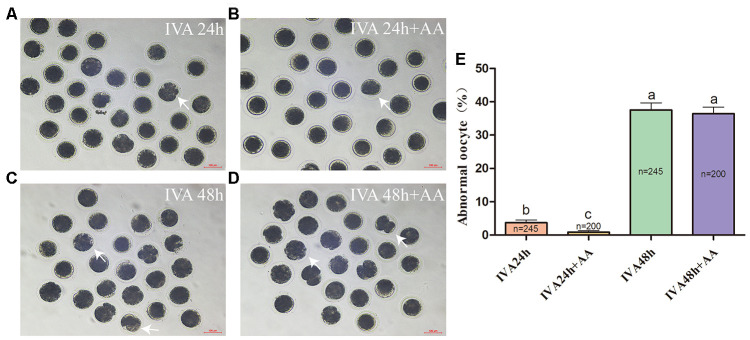
**Changes in the morphology of *in vitro* aged porcine oocytes after AA supplementation.** (**A**) 24-h *in vitro* aged oocytes; (**B**) 24-h *in vitro* aged oocytes supplemented with 10 μM AA; (**C**) 48-h *in vitro* aged oocytes; (**D**) 48-h *in vitro* aged oocytes supplemented with 10 μM AA; (**E**) morphological changes in porcine oocytes recovered at different culture time points from the AA supplementation groups. Representative morphologically abnormal *in vitro* aged oocytes (white arrows) were examined by optical microscopy. Scale bar=100 μm. The numbers of oocytes examined in each group are shown by the bars. Statistically significant differences are indicated by different letters (*p<0.05*).

Abnormal morphology often results in morphological changes. Therefore, we first investigated whether AA supplementation promoted the maintenance of the morphology of aged porcine oocytes. After *in vitro* maturation (IVM), mature oocytes were collected for IVA, and morphological changes were recorded at 24 and 48 h (Figure 1A–1D). As shown in Figure 1E, the percentage of abnormal oocytes increased in a time-dependent manner (IVA 24 h, 3.70 ± 0.81%, n=245; IVA 48 h, 37.52 ± 2.17%, n=245; *p<0.05*). Compared with the non-AA-supplemented group, the group of *in vitro* aged porcine oocytes supplemented with 10 μM AA showed a decrease in the percentage of oocytes with abnormal morphology at 24 h (0.85 ± 0.50%, n=200; 3.70 ± 0.81%, n=245; *p<0.05*). However, the morphological defects were not dramatically reduced by AA supplementation at 48 h of IVA (36.41 ± 1.97%, n=200; 37.52 ± 2.17%, n=245; *p>0.05*).

### AA supplementation improves the embryonic developmental potential of porcine *in vitro* aged oocytes after parthenogenetic activation

**Figure 2:**
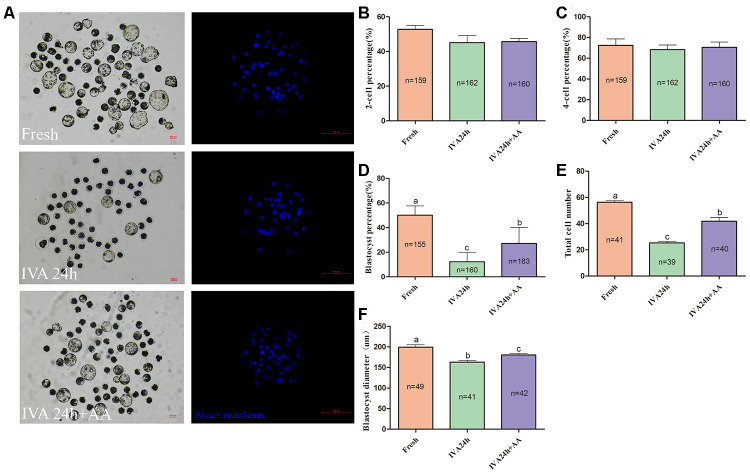
**Effect of AA supplementation on the developmental competence of *in vitro* aged porcine oocytes after parthenogenetic activation.** (**A**) Blastocyst formation at day 7 in each group (Left); and representative fluorescence images of blastocyst staining with Hoechst 33342 in each group (Right). Scale bar=100 μm. (**B**) Percentage of 2-cell embryos, (**C**) 4-cell embryos and (**D**) blastocyst formation in each group. (**E**) Total number of cells in blastocysts from each group. (**F**) Diameter of blastocysts from each group. The number of embryos examined in each group is shown by the bars. Statistically significant differences are indicated by different letters (*p<0.05*).

To extend our finding that supplementation with AA ameliorated the time-dependent deterioration of porcine oocyte quality, oocytes were parthenogenetically activated to evaluate subsequent embryonic development (Figure 2A). The results showed that AA supplementation during the IVA process ameliorated the subsequent declines in developmental competence. Although the percentage of activated oocytes that reached the 2-cell stage (52.77 ± 2.25%, n=159; 45.18 ± 4.00%, n=162; 45.76 ± 1.78%, n=160; *p>0.05*; Figure 2B) and 4-cell stage (72.25 ± 6.42%, n=159; 68.37 ± 4.36%, n=162; 70.50 ± 5.14%, n=160; *p>0.05*; Figure 2C) showed no obvious change, when *in vitro* aged porcine oocytes were supplemented with AA, the percentage of activated oocytes that reached the blastocyst stage was obviously improved compared with that not supplemented with AA (50.19 ± 2.85%, n=155; 12.27 ± 2.91%, n=160; 27.19 ± 4.94%, n=183; *p<0.05*; Figure 2D). Furthermore, the average total number of cells in blastocysts derived from AA-supplemented *in vitro* aged oocytes tended to be higher than that in blastocysts derived from non-supplemented *in vitro* aged oocytes (56.24 ± 1.13, n=41; 25.26 ± 1.18, n=39; 41.75 ± 2.96, n=40; *p<0.05*; Figure 2E). The average diameter of blastocysts derived from AA-supplemented *in vitro* aged oocytes was also decreased compared with that derived from non-supplemented *in vitro* aged oocytes (199.57 ± 5.86, n=49; 163.10 ± 4.18, n=41;180.31 ± 3.51, n=42; *p<0.05*; Figure 2F).

### AA supplementation ameliorates sperm the binding ability of porcine *in vitro* aged oocytes and subsequent embryonic developmental competence after *in vitro* fertilization

**Figure 3:**
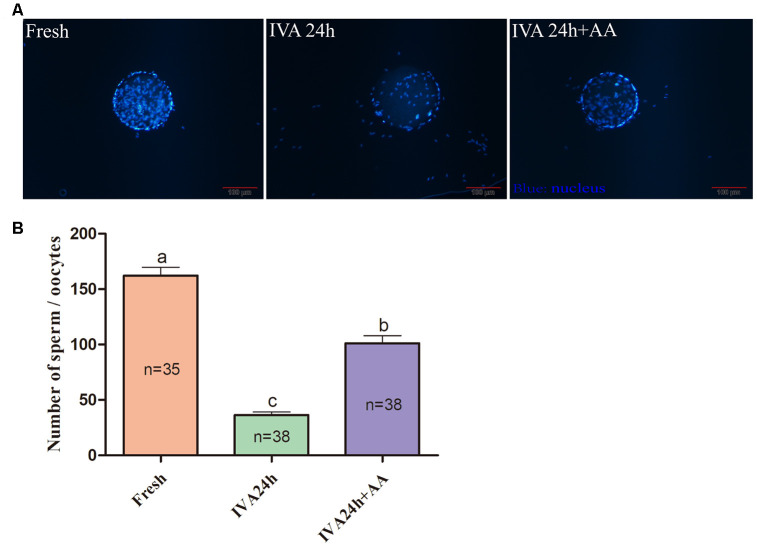
**Effect of AA supplementation on sperm binding to the zona pellucida of porcine *in vitro* aged oocytes after *in vitro* fertilization.** (**A**) Representative fluorescence images of sperm binding to the surface of the zona pellucida surrounding oocytes staining with Hoechst 33342 in each group. Scale-bar=100 μm. (**B**) Number of sperm binding to the surface of the zona pellucida surrounding oocytes in each group. The number of oocytes examined in each group is shown by the bars. Statistically significant differences are indicated by different letters (*p<0.05*).

**Figure 4:**
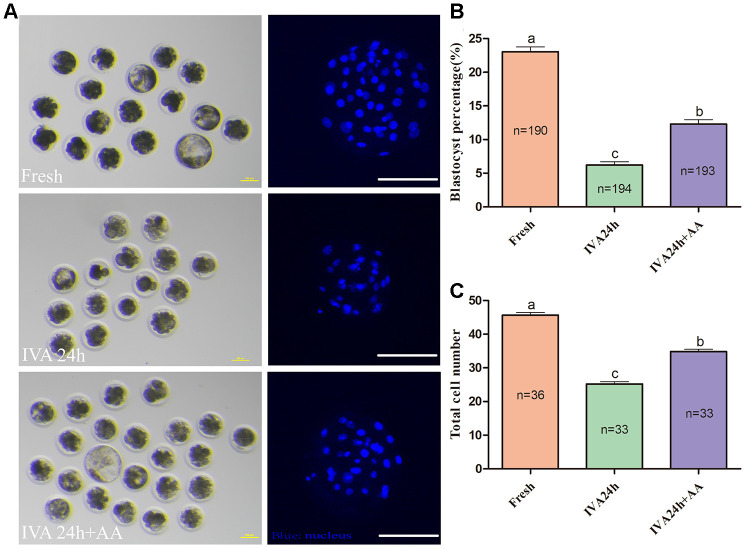
**Effect of AA supplementation on the developmental competence of porcine *in vitro* aged oocytes after *in vitro* fertilization.** (**A**) Blastocyst formation at day 7 in each group (Left); and representative fluorescence images of blastocyst staining with Hoechst 33342 in each group (Right). Scale bar=100 μm. (**B**) Percentage of blastocyst formation in each group. (C) Total number of cells in blastocysts from each group. The number of embryos examined in each group is shown by the bars. Statistically significant differences are indicated by different letters (*p<0.05*).

The results of the present research showed that the decreased oocyte developmental competence induced by IVA was ameliorated by AA supplementation after *in vitro* fertilization. Sperm-egg binding assay results suggested that the average number of sperm that bound to the zona pellucida of *in vitro* aged oocytes tended to be higher in the AA-supplemented group than the non-supplemented *in vitro* aged group (162.26 ± 7.53, n=35; 36.34 ± 2.85, n=38; 101.28 ± 6.83, n=38; *p<0.05*; Figure 3A and 3B). The above results showed that AA supplementation could improve the quality of porcine oocytes. Therefore, *in vitro* fertilization was carried out with fresh, *in vitro* aged and AA-supplemented *in vitro* aged porcine oocytes (Figure 4A). The results showed that 23.06 ± 0.72% (n=190) of fresh oocytes could develop into the blastocyst stage. For *in vitro* aged porcine oocytes, the percentage of oocytes that developed into the blastocyst stage decreased to 6.23 ± 0.48% (n=194). However, in the AA-supplemented *in vitro* aged oocyte group, the percentage of oocytes that developed into the blastocyst stage was significantly increased (12.30 ± 0.67%, n=193). Similar to the blastocyst formation results, the average total number of cells in blastocysts derived from AA-supplemented *in vitro* aged oocytes was also increased compared with that of blastocysts derived from non-supplemented *in vitro* aged oocytes (45.68 ± 0.76, n=36; 25.22 ± 0.71, n=33; 34.85 ± 0.67, n=33; *p<0.05*; Figure 4C).

### AA supplementation ameliorates oxidation resistance in porcine IVA oocytes

**Figure 5:**
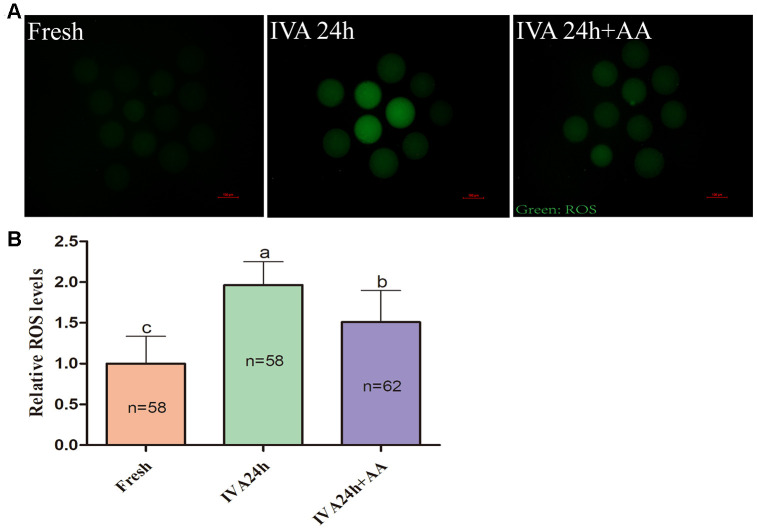
**Effects of AA supplementation on the production of intracellular ROS in porcine IVA oocytes.** (**A**) Representative fluorescence images of intracellular ROS in oocytes in each group. Scale bar=100 μm. (**B**) Relative intracellular ROS levels in oocytes in each group. The number of oocytes examined in each group is shown by the bars. Statistically significant differences are indicated by different letters (*p<0.05*).

**Figure 6:**
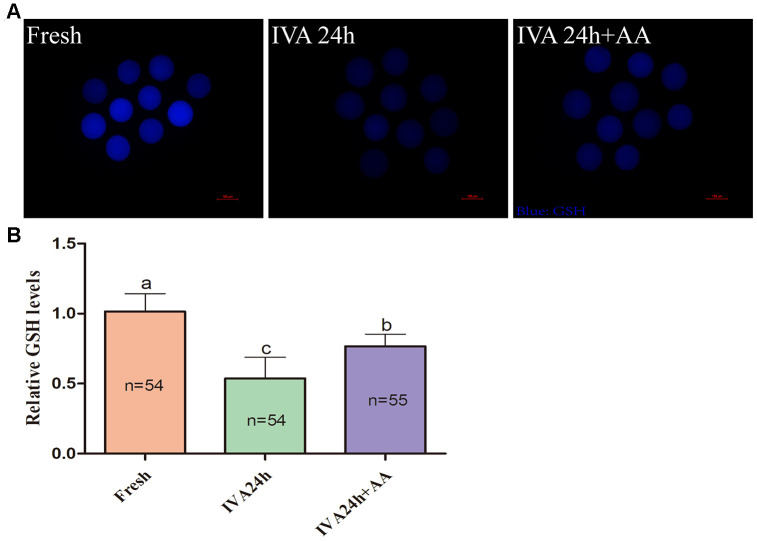
**Effects of AA supplementation on the production of intracellular GSH in porcine IVA oocytes.** (**A**) Representative fluorescence images of intracellular GSH in oocytes in each group. Scale bar=100 μm. (**B**) Relative intracellular GSH levels in oocytes in each group. The number of oocytes examined in each group is shown by the bars. Statistically significant differences are indicated by different letters (*p<0.05*).

Because oxidative stress was associated with oocyte quality and subsequent embryonic developmental competence, we further assessed the free radical scavenging capacity of AA in *in vitro* aged porcine oocytes. The DCFH fluorescent reaction was used to analyze intracellular ROS levels in oocytes. As shown in Figure 5A and 5B, intracellular ROS levels were markedly increased in *in vitro* aged porcine oocytes compared with fresh oocytes, while AA supplementation effectively alleviated the increase in intracellular ROS levels during the IVA process of porcine oocytes. In accordance with the above results, we further assessed the intracellular GSH levels of *in vitro* aged porcine oocytes. As shown in Figure 6A and 6B, the intracellular GSH levels of *in vitro* aged porcine oocytes were markedly decreased compared with those of fresh oocytes, while AA supplementation effectively alleviated the decline in intracellular GSH levels in porcine oocytes during the IVA process.

### AA supplementation decreased the early apoptosis of *in vitro* aged porcine oocytes

**Figure 7:**
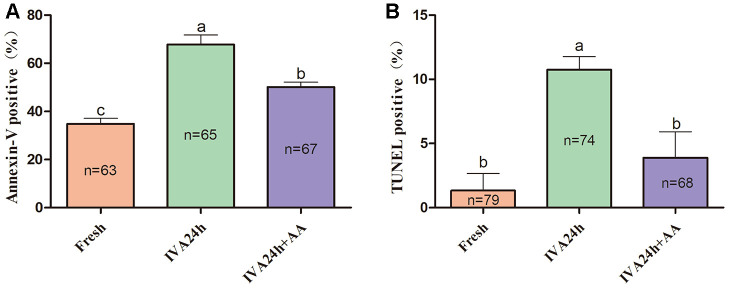
**Effects of AA supplementation on the early apoptosis of *in vitro* aged porcine oocytes.** (**A**) Percentage of Annexin-V and (**B**) TUNEL positive oocytes in each group. The number of oocytes examined in each group is shown by the bars. Statistically significant differences are indicated by different letters (*p<0.05*).

To further define the role of AA during the IVA process of porcine oocytes, the early apoptosis of *in vitro* aged porcine oocytes was monitored via the Annexin-V and TUNEL assays. As shown in Figure 7A and 7B, only 34.84 ± 2.26% (n=63) and 1.33 ± 1.33% (n=79) of fresh oocytes were positive for Annexin-V and TUNEL, respectively; however, the proportion of Annexin-V and TUNEL positive oocytes among *in vitro* aged oocytes were increased to 67.78 ± 3.98% (n=65) and 10.74 ± 1.03%, respectively (n=74); *p<0.05*. In contrast, in oocytes supplemented with AA during the process of IVA, the proportion of Annexin-V and TUNEL positive oocytes was reduced to 50.14 ± 2.00% (n=67) and 3.89 ± 2.04%, respectively (n=68); *p<0.05*.

### AA supplementation alleviated mitochondrial function in porcine IVA oocytes

**Figure 8:**
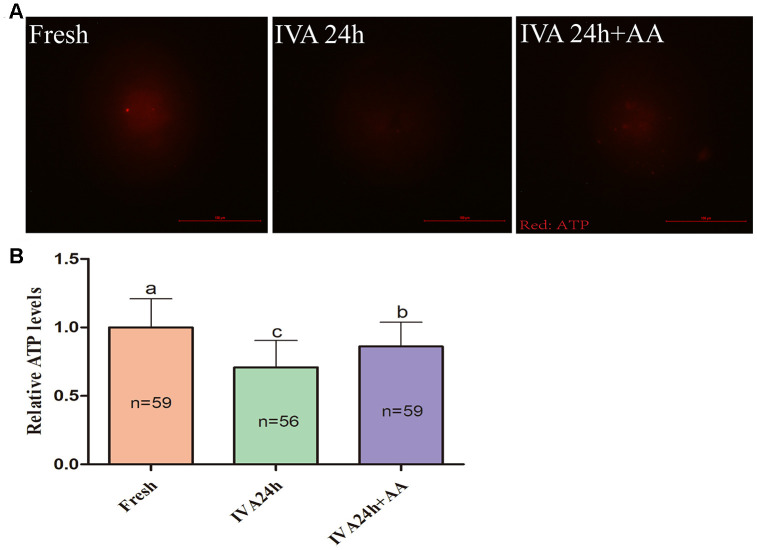
**Effects of AA supplementation on the ATP production of *in vitro* aged porcine oocytes.** (**A**) Representative fluorescence images of ATP staining in oocytes from each group. Scale bar=100 μm. (**B**) Relative ATP levels in oocytes from each group. The number of oocytes examined in each group is shown by the bars. Statistically significant differences are indicated by different letters (*p<0.05*).

**Figure 9:**
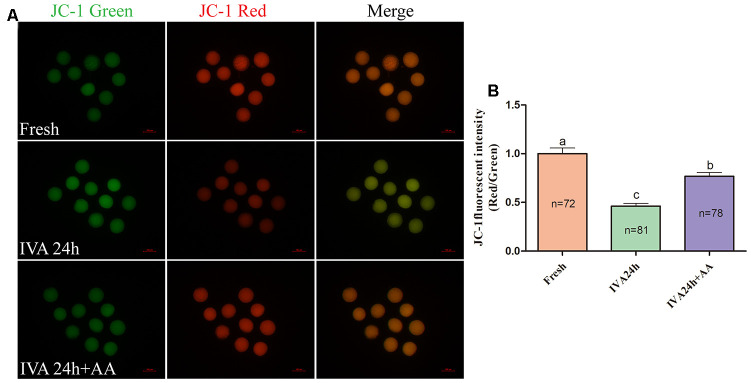
**Effects of AA supplementation on the mitochondrial function of *in vitro* aged porcine oocytes.** (**A**) Representative fluorescent images of JC-1 stained oocytes from each group. Scale bar=100 μm. (**B**) Relative JC-1 fluorescence intensity (red/green) in oocytes from each group. The number of oocytes examined in each group is shown by the bars. Statistically significant differences are indicated by different letters (*p<0.05*).

Mitochondria play important roles in the regulation of cellular energy metabolism, free radical generation and apoptosis. Therefore, we evaluated ATP levels and mitochondrial membrane potential (MMP) in porcine IVA oocytes. As shown in Figure 8A and 8B, the ATP levels of *in vitro* aged porcine oocytes were markedly decreased compared with those fresh oocytes, while AA supplementation effectively alleviated the decline in ATP levels during the IVA process of porcine oocytes. The MMP of *in vitro* aged porcine oocytes was evaluated using the JC-1 fluorescent dye (Figure 9A). Quantification showed that the MMP of *in vitro* aged porcine oocytes was markedly decreased compared with that of fresh oocytes, while AA supplementation effectively alleviated the decrease in MMP during the IVA process of porcine oocytes (Figure 9B).

## DISCUSSION

The IVM of pig oocytes is used not only for understanding the event of meiosis but also for helping breed, clone and genome edit pigs for animal production. Previous research has shown that compared with that of other species oocytes, the maturation time of pig oocytes is prolonged [35]. During IVM, porcine oocytes are particularly susceptible to aging, leading to disturbances in metabolism, stress response and cell cycle regulation [36]. These changes reduce the quality of oocytes [13] and impact later embryonic development [7, 37]. In the present research, we found that AA could delay the process of pig oocyte IVA and subsequently improve embryonic developmental potential. Fertilization is a complex process of sexual reproduction in which the female and male gametes are mixed to generate new individuals [38]. Therefore, fertilization ability is one of the indicators used to evaluate oocyte quality. Our results show that AA can alleviate the decreased fertilization ability of aged oocytes, which is consistent with our results that AA could improve the parthenogenetic activation of developing embryos. In the reproduction process of animals, a particular sperm needs to arrive at, attach to, and mix with the egg plasma membrane; hence, it has to attach to and pass through the zona pellucida [39–41]. In other words, fertilization begins with the binding of the sperm to the zona pellucida. We found that AA could increase the number of sperm that bind to aged oocytes. Early studies suggested that the process of oocyte aging is usually accompanied by zona pellucida changes [5]. Thus, we suspect that AA may have other potential mechanisms to mitigate fertility declines due to aging by altering certain components of the zona pellucida.

Previous studies have shown that the quality of oocytes decreases gradually during the IVA process and that aged oocytes are sensitive to oxidative stress [37, 42]. Thus, this process of oocyte aging easily results in oxidative stress via the accumulation of free radicals. The increase in intracellular ROS could reduce the developmental potential of oocytes [43]. Mammalian cells have a wide range of antioxidants that have the ability to clear ROS, including non-enzymatic antioxidants [44] and several enzymatic antioxidants [45]. GSH, as an intrinsic antioxidant in oocytes, plays an important role in maintaining the redox state in oocytes [46]. GSH deficiency could lead to increased intracellular oxidative stress [47], germ cell apoptosis [48], and widespread mitochondrial damage [49], and a high intracellular GSH content could improve the quality of the oocytes [50]. In addition, a lack of GSH synthesis is the basis of oxidative stress during aging [51]. Our results demonstrate that AA supplementation clearly ameliorated oxidative stress because it not only decreased intracellular ROS levels but also maintained intracellular GSH levels during the process of IVA. Similarly, AA was reported to reduce intracellular ROS levels in microglial cells [52], HepG2 cells [53], and SH-SYS5Y cells [54] and increase superoxide dismutase activity and intracellular GSH production in spinal cord injury in rats [55]. Therefore, we hypothesized that AA might reduce oxidative stress by removing intracellular ROS and increasing GSH synthesis, thus delaying the decline in porcine oocyte quality during the process of IVA.

Functional mitochondria play an important role in normal oocyte function, since within both oocytes and early embryos, these organelles are the major source of ATP production [56]. Proton pumps, expressed on both sides of the mitochondrial membrane, regulate the potential of the mitochondrial membrane by the production of ATP [57]. An insufficient supply of ATP affects oocyte quality, leading to many problems, such as arrested cell division, abnormal cytokinesis, fragmentation [58–60], maturation and fertilization failure [17]. In mammals, the number of mitochondria increases dramatically with the growth of oocytes [61, 62], and after fertilization, the number of mitochondria remains constant from the early stages of embryo development through embryo implantation [62, 63]. Furthermore, abnormal MMP is closely related to fertilization, oocyte maturation, calcium homeostasis maintenance and apoptosis [17, 37, 64]. Therefore, it is very important for oocytes to have sufficient properly functioning mitochondria to ensure appropriate embryonic development. Increasing data have proven that changes in mitochondrial function are inextricably linked with stress-induced damage to oocyte developmental capacity [65]. Recent research shows that this impairment decreases mitochondrial ROS production, impairs the ability of mitochondria to sufficiently meet the cellular ATP demand, and induces mitochondrial-mediated apoptosis [66]. Moreover, mitochondria are considered to be a key element in the process of oocyte aging, which is greatly impacted by mitochondrial dysfunction [67, 68]. Our study shows that AA reduced the level of intracellular ROS and increased ATP activity and the red/green fluorescence ratio of JC-1, suggesting that mitochondrial dysfunction and oxidative stress were improved by AA supplementation during the process of porcine oocyte IVA. Since the findings in these experiments are in line with previous studies, it can be concluded that mitochondrial dysfunction can be well reduced by AA in various cells, including murine BV2 microglia cells, rat cortical neuronal cells [69, 70] and human A549 cells [71], and these results further confirm our hypothesis.

Since cell death via apoptosis is the last stage of oocyte aging [72, 73], it is hypothesized that AA can inhibit the activation of the apoptotic pathway of aged oocytes. We found that AA could delay the commencement of apoptosis in aged oocytes by Annexin-V and TUNEL assays. This function of AA is consistent with the results of other studies, which highlighted that AA protects against lactate-induced apoptosis through controlling the lactate signaling cascade and mediating mitochondria-dependent caspase activation and oxidative stress [71]; moreover, because of its antioxidant, mito-protective and antiapoptotic properties, pretreatment with AA before rotenone exposure in SH-SY5Y cells prevented marked ROS overproduction, mitochondrial dysfunction and apoptosis [54]. In early studies, the aging of humans and mammals was shown to be closely related to an increased level of ROS in mitochondria with age, which subsequently triggered apoptosis [74]. The problems associated with IVA appear to have a similar mechanism to the body's response to aging, which needs further study.

**Figure 10:**
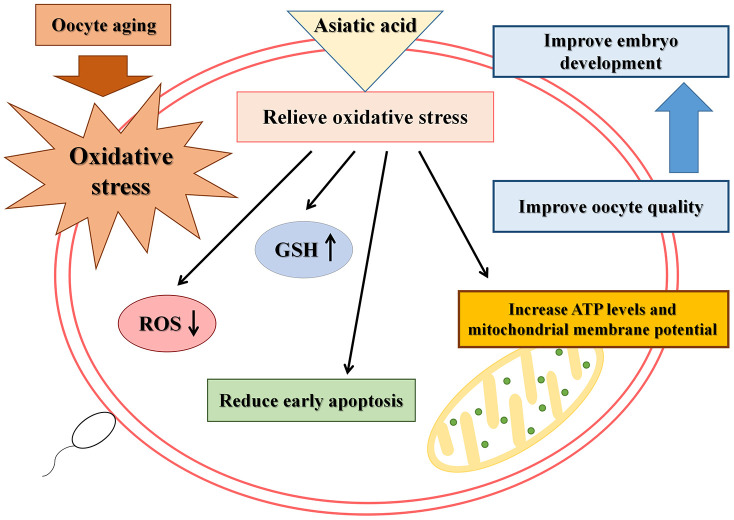
**Schematic representation of the protective action of AA on porcine IVA oocytes.**

In summary, we found that AA could effectively improve the quality of *in vitro* aged porcine oocytes by relieving oxidative stress, reducing apoptosis, improving mitochondrial function, and promoting subsequent embryonic development (Figure 10). In other words, as an outstanding antioxidant that can be extracted from natural products, AA is expected to prevent the *in vitro* aging-induced decrease in oocyte quality.

## MATERIALS AND METHODS

All chemicals used in this experiment were obtained from Sigma-Aldrich, a company based in St. Louis, America, except for those that are specifically defined.

### Oocyte collection and IVM

Porcine ovaries were collected from a local abattoir and transported to the laboratory in 0.9% saline at 30-35° C. Follicular fluid was aspirated from 3-8 mm antral follicles using a syringe needle. Only evenly distributed cumulus oocyte complexes (COCs) with more than three layers of cumulus cells were collected under a stereomicroscope (S22-LGB, Nikon). After that, the COCs were washed three times in Tyrode’s lactate-HEPES buffered medium and were cultured in IVM medium (tissue culture medium 199 supplemented with 0.6 mM L-cysteine, 0.91 mM Na pyruvate, 10 ng/mL EGF, 10% porcine follicular fluid, 10 IU/mL follicle-stimulating hormone, 10 IU/mL luteinizing hormone and 75 mg/mL kanamycin) for 42 h at 38.5° C in an atmosphere of 5% CO_2_ and 100% humidity covered with mineral oil.

### IVA of porcine oocytes

At the end of IVM, the expanded cumulus cells were removed from COCs by treatment with 0.1% hyaluronidase, and only matured oocytes with normal morphology were collected for IVA. The oocytes were then cultured in IVM medium, treated or not treated with 10 μM AA, covered with mineral oil in a damp environment of 38.5° C and 5% CO_2_ and cultured for 24 or 48 h. The final concentration of AA used in the present research was selected according to our preliminary experiment.

### Parthenogenetic activation and *in vitro* fertilization

The parthenogenetic activation and *in vitro* fertilization procedures were performed as described in our previous research [33]. After parthenogenetic activation and *in vitro* fertilization, activated or fertilized oocytes were washed in porcine zygote medium (PZM)-3 and then transferred into four-well plates containing 500 μL PZM-3 covered with mineral oil and cultured for 7 days at 38.5° C in an atmosphere of 5% CO_2_ and 100% humidity without changing the medium. Blastocyst rates and diameters were analyzed on day 7, and blastocyst diameters were analyzed via NIH ImageJ software (National Institutes of Health, Bethesda, MD, USA).

### Assessment of the total number of cells in blastocysts

To quantifying the total number of cells contained in blastocysts, day 7 blastocysts derived from embryos that underwent parthenogenetic activation and *in vitro* fertilization were fixed in PBS-PVA with 4% PFA for 30 min at room temperature. Then, the blastocysts were washed with PBS-PVA three times and incubated with 10 μg/mL Hoechst 33342 for 5 min to label the nuclei. Next, blastocysts were gently mounted onto glass slides and examined under a fluorescence microscope (IX73, Olympus, Tokyo, Japan). NIH ImageJ software was used to analyze the total number of nuclei.

### Sperm binding assay

Frozen boar sperm samples were used in this research, and the percentage of motile spermatozoa after thawing was 70%-90%. Approximately 15-20 oocytes were transferred into 60 μl droplets of mTBM for 30 min and then co-incubated with 20 μl diluted sperm for 1 h at 38.5° C in an atmosphere of 5% CO_2_ and 100% humidity. Afterwards, the oocytes were fixed in PBS-PVA with 4% PFA for 30 min at room temperature and stained with 10 μg/mL Hoechst 33342. Next, the oocytes were gently mounted onto glass slides and examined under a fluorescence microscope. The number of sperm bound to each oocyte was analyzed via NIH ImageJ software.

### Evaluation of intracellular ROS and GSH levels in oocytes

The intracellular ROS and GSH levels in fresh and aged oocytes were measured using the fluorescent probes 2',7’-dichlorodihydrofluorescein diacetate (10 μM, Thermo Fisher Scientific, C400) and 4-chloromethyl-6,8-difluoro-7-hydroxycoumarin (10 μM, Thermo Fisher Scientific, C12881), respectively. Oocytes from each treatment group were incubated in the dark for 25 min at 38.5° C in an atmosphere of 5% CO_2_ and 100% humidity. After incubation, the oocytes were washed three times with PBS-PVA, and 10 μL droplets were removed. The fluorescence signal was captured using an epifluorescence microscope, and the fluorescence intensity of oocytes was analyzed using NIH ImageJ software.

### Evaluation of oocyte apoptosis

The apoptosis of fresh and aged oocytes was measured via Annexin-V and TUNEL assays. Staining procedures were performed according to the product manual. For the Annexin-V assay, the oocytes were incubated in 100 μL Annexin-V binding solution with 5 μL Annexin-V and fluorescein isothiocyanate (FITC) conjugate for 30 min in the dark at room temperature. For the TUNEL assay, the oocytes were incubated with 50 μL TUNEL reaction mixture for 1 h at 37° C in the dark. The fluorescence signal was captured using a confocal microscope.

### ATP measurement in oocytes

The ATP content of individual oocytes was measured using a fluorescent antibody (Abcam, 198367). Briefly, oocytes were collected, washed twice in PBS-PVA and then fixed in PBS-PVA with 4% PFA for 10 min at room temperature. Afterwards, the oocytes were washed three times with ice-cold PBS-PVA and then incubated with PBS-PVA containing 0.1% Triton X-100 for membrane permeabilization for 1 h. After being blocked for 30 min in 1.5% bovine serum albumin prepared in PBS-PVA (blocking solution), the oocytes were incubated with the antibody diluted in blocking solution (1:100, v/v) overnight at 4° C. After thoroughly washing with PBS-PVA, the fluorescence signal was captured using an epifluorescence microscope, and the fluorescence intensity of oocytes was analyzed using NIH ImageJ software.

### JC-1 staining

To measure MMP, fresh and aged oocytes were washed three times with PBS-PVA. They were measured using the fluorescent probe 5,5’,6,6’-tetrachloro-1,1’,3,3’-tetraethyl-imidacarbocyanine iodide (2 μM, JC-1, Dojindo, China) for 30 min. After incubation, the oocytes were washed three times with the solution in the kit. The red and green fluorescence signals were captured using a fluorescence microscope connected to a digital camera. The fluorescence intensity of each oocyte was analyzed using NIH ImageJ software.

### Statistical analysis

SPSS software version 11.0 (IBM, USA) was used to analyze all the data collected. The data from the two groups were compared with Student's t-test, the results are presented as the mean ± standard error of the mean, and considerable discrepancies are indicated by different letters (*p*<0.05).

## References

[1] Davies MJ, Rumbold AR, Moore VM. Assisted reproductive technologies: a hierarchy of risks for conception, pregnancy outcomes, and treatment decisions - ERRATUM. J Dev Orig Health Dis. 2018; 9:241–46. 10.1017/S2040174417000757 28978364

[2] Kocourkova J, Burcin B, Kucera T. Demographic relevancy of increased use of assisted reproduction in European countries. Reprod Health. 2014; 11:37. 10.1186/1742-4755-11-37 24885428 PMC4049397

[3] Sunderam S, Kissin DM, Zhang Y, Folger SG, Boulet SL, Warner L, Callaghan WM, Barfield WD. Assisted reproductive technology surveillance - United States, 2016. MMWR Surveill Summ. 2019; 68:1–23. 10.15585/mmwr.ss6804a1 31022165 PMC6493873

[4] Lord T, Aitken RJ. Oxidative stress and ageing of the post-ovulatory oocyte. Reproduction. 2013; 146:R217–27. 10.1530/REP-13-0111 23950493

[5] Miao YL, Kikuchi K, Sun QY, Schatten H. Oocyte aging: cellular and molecular changes, developmental potential and reversal possibility. Hum Reprod Update. 2009; 15:573–85. 10.1093/humupd/dmp014 19429634

[6] Takahashi T, Igarashi H, Amita M, Hara S, Matsuo K, Kurachi H. Molecular mechanism of poor embryo development in postovulatory aged oocytes: mini review. J Obstet Gynaecol Res. 2013; 39:1431–39. 10.1111/jog.12111 23876057

[7] Lin T, Lee JE, Kang JW, Oqani RK, Cho ES, Kim SB, Il Jin D. Melatonin supplementation during prolonged *in vitro* maturation improves the quality and development of poor-quality porcine oocytes via anti-oxidative and anti-apoptotic effects. Mol Reprod Dev. 2018; 85:665–81. 10.1002/mrd.23052 30106229

[8] Kim E, Jeon Y, Kim DY, Lee E, Hyun SH. Antioxidative effect of carboxyethylgermanium sesquioxide (Ge-132) on IVM of porcine oocytes and subsequent embryonic development after parthenogenetic activation and IVF. Theriogenology. 2015; 84:226–36. 10.1016/j.theriogenology.2015.03.006 25913277

[9] Xu Z, Abbott A, Kopf GS, Schultz RM, Ducibella T. Spontaneous activation of ovulated mouse eggs: time-dependent effects on m-phase exit, cortical granule exocytosis, maternal messenger ribonucleic acid recruitment, and inositol 1,4,5-trisphosphate sensitivity. Biol Reprod. 1997; 57:743–50. 10.1095/biolreprod57.4.743 9314575

[10] Wilding M, Dale B, Marino M, di Matteo L, Alviggi C, Pisaturo ML, Lombardi L, De Placido G. Mitochondrial aggregation patterns and activity in human oocytes and preimplantation embryos. Hum Reprod. 2001; 16:909–17. 10.1093/humrep/16.5.909 11331637

[11] Wakayama S, Thuan NV, Kishigami S, Ohta H, Mizutani E, Hikichi T, Miyake M, Wakayama T. Production of offspring from one-day-old oocytes stored at room temperature. J Reprod Dev. 2004; 50:627–37. 10.1262/jrd.50.627 15647614

[12] Steuerwald NM, Steuerwald MD, Mailhes JB. Post-ovulatory aging of mouse oocytes leads to decreased MAD2 transcripts and increased frequencies of premature centromere separation and anaphase. Mol Hum Reprod. 2005; 11:623–30. 10.1093/molehr/gah231 16207798

[13] Takahashi T, Takahashi E, Igarashi H, Tezuka N, Kurachi H. Impact of oxidative stress in aged mouse oocytes on calcium oscillations at fertilization. Mol Reprod Dev. 2003; 66:143–52. 10.1002/mrd.10341 12950101

[14] Mihalas BP, Redgrove KA, McLaughlin EA, Nixon B. Molecular mechanisms responsible for increased vulnerability of the ageing oocyte to oxidative damage. Oxid Med Cell Longev. 2017; 2017:4015874. 10.1155/2017/4015874 29312475 PMC5664291

[15] Lee SE, Kim EY, Choi HY, Moon JJ, Park MJ, Lee JB, Jeong CJ, Park SP. Rapamycin rescues the poor developmental capacity of aged porcine oocytes. Asian-Australas J Anim Sci. 2014; 27:635–47. 10.5713/ajas.2013.13816 25049998 PMC4093196

[16] Lord T, Nixon B, Jones KT, Aitken RJ. Melatonin prevents postovulatory oocyte aging in the mouse and extends the window for optimal fertilization *in vitro*. Biol Reprod. 2013; 88:67. 10.1095/biolreprod.112.106450 23365415

[17] Van Blerkom J. Mitochondrial function in the human oocyte and embryo and their role in developmental competence. Mitochondrion. 2011; 11:797–813. 10.1016/j.mito.2010.09.012 20933103

[18] Liu L, Trimarchi JR, Navarro P, Blasco MA, Keefe DL. Oxidative stress contributes to arsenic-induced telomere attrition, chromosome instability, and apoptosis. J Biol Chem. 2003; 278:31998–2004. 10.1074/jbc.M303553200 12767976

[19] Sies H. Strategies of antioxidant defense. Eur J Biochem. 1993; 215:213–19. 10.1111/j.1432-1033.1993.tb18025.x 7688300

[20] Sies H. Oxidative stress: oxidants and antioxidants. Exp Physiol. 1997; 82:291–95. 10.1113/expphysiol.1997.sp004024 9129943

[21] Lv J, Sharma A, Zhang T, Wu Y, Ding X. Pharmacological review on Asiatic acid and its derivatives: a potential compound. SLAS Technol. 2018; 23:111–27. 10.1177/2472630317751840 29361877

[22] Ren L, Cao QX, Zhai FR, Yang SQ, Zhang HX. Asiatic acid exerts anticancer potential in human ovarian cancer cells via suppression of PI3K/Akt/mTOR signalling. Pharm Biol. 2016; 54:2377–82. 10.3109/13880209.2016.1156709 26984021

[23] Ding H, Xiong Y, Sun J, Chen C, Gao J, Xu H. Asiatic acid prevents oxidative stress and apoptosis by inhibiting the translocation of α-synuclein into mitochondria. Front Neurosci. 2018; 12:431. 10.3389/fnins.2018.00431 30002614 PMC6031891

[24] Qian Y, Xin Z, Lv Y, Wang Z, Zuo L, Huang X, Li Y, Xin HB. Asiatic acid suppresses neuroinflammation in BV2 microglia via modulation of the Sirt1/NF-κB signaling pathway. Food Funct. 2018; 9:1048–57. 10.1039/c7fo01442b 29354820

[25] Dong SH, Liu YW, Wei F, Tan HZ, Han ZD. Asiatic acid ameliorates pulmonary fibrosis induced by bleomycin (BLM) via suppressing pro-fibrotic and inflammatory signaling pathways. Biomed Pharmacother. 2017; 89:1297–309. 10.1016/j.biopha.2017.03.005 28320097

[26] Ahmad Rather M, Justin Thenmozhi A, Manivasagam T, Dhivya Bharathi M, Essa MM, Guillemin GJ. Neuroprotective role of Asiatic acid in aluminium chloride induced rat model of Alzheimer’s disease. Front Biosci (Schol Ed). 2018; 10:262–75. 10.2741/s514 28930532

[27] Sun W, Xu G, Guo X, Luo G, Wu L, Hou Y, Guo X, Zhou J, Xu T, Qin L, Fan Y, Han L, Matsabisa M, et al. Protective effects of Asiatic acid in a spontaneous type 2 diabetic mouse model. Mol Med Rep. 2017; 16:1333–39. 10.3892/mmr.2017.6684 28586016 PMC5562101

[28] Wang X, Lu Q, Yu DS, Chen YP, Shang J, Zhang LY, Sun HB, Liu J. Asiatic acid mitigates hyperglycemia and reduces islet fibrosis in Goto-Kakizaki rat, a spontaneous type 2 diabetic animal model. Chin J Nat Med. 2015; 13:529–34. 10.1016/S1875-5364(15)30047-9 26233843

[29] Maneesai P, Bunbupha S, Kukongviriyapan U, Senggunprai L, Kukongviriyapan V, Prachaney P, Pakdeechote P. Effect of Asiatic acid on the Ang II-AT_1_R-NADPH oxidase-NF-κB pathway in renovascular hypertensive rats. Naunyn Schmiedebergs Arch Pharmacol. 2017; 390:1073–83. 10.1007/s00210-017-1408-x 28733880

[30] Loganathan C, Thayumanavan P. Asiatic acid prevents the quinolinic acid-induced oxidative stress and cognitive impairment. Metab Brain Dis. 2018; 33:151–59. 10.1007/s11011-017-0143-9 29086235

[31] Wang ZH, Mong MC, Yang YC, Yin MC. Asiatic acid and maslinic acid attenuated kainic acid-induced seizure through decreasing hippocampal inflammatory and oxidative stress. Epilepsy Res. 2018; 139:28–34. 10.1016/j.eplepsyres.2017.11.003 29156327

[32] Lv H, Qi Z, Wang S, Feng H, Deng X, Ci X. Asiatic acid exhibits anti-inflammatory and antioxidant activities against lipopolysaccharide and d-galactosamine-induced fulminant hepatic failure. Front Immunol. 2017; 8:785. 10.3389/fimmu.2017.00785 28736552 PMC5500632

[33] Qi JJ, Li XX, Diao YF, Liu PL, Wang DL, Bai CY, Yuan B, Liang S, Sun BX. Asiatic acid supplementation during the *in vitro* culture period improves early embryonic development of porcine embryos produced by parthenogenetic activation, somatic cell nuclear transfer and *in vitro* fertilization. Theriogenology. 2020; 142:26–33. 10.1016/j.theriogenology.2019.09.027 31574397

[34] Chao PC, Yin MC, Mong MC. Anti-apoptotic and anti-glycative effects of Asiatic acid in the brain of D-galactose treated mice. Food Funct. 2015; 6:542–48. 10.1039/c4fo00862f 25504333

[35] Kalous J, Tetkova A, Kubelka M, Susor A. Importance of ERK1/2 in regulation of protein translation during oocyte meiosis. Int J Mol Sci. 2018; 19:698. 10.3390/ijms19030698 29494492 PMC5877559

[36] Jiang GJ, Wang K, Miao DQ, Guo L, Hou Y, Schatten H, Sun QY. Protein profile changes during porcine oocyte aging and effects of caffeine on protein expression patterns. PLoS One. 2011; 6:e28996. 10.1371/journal.pone.0028996 22194971 PMC3241687

[37] Wang T, Gao YY, Chen L, Nie ZW, Cheng W, Liu X, Schatten H, Zhang X, Miao YL. Melatonin prevents postovulatory oocyte aging and promotes subsequent embryonic development in the pig. Aging (Albany NY). 2017; 9:1552–64. 10.18632/aging.101252 28657543 PMC5509455

[38] Ohto U, Ishida H, Krayukhina E, Uchiyama S, Inoue N, Shimizu T. Structure of IZUMO1-JUNO reveals sperm-oocyte recognition during mammalian fertilization. Nature. 2016; 534:566–69. 10.1038/nature18596 27309808

[39] Clark GF. The molecular basis of mouse sperm-zona pellucida binding: a still unresolved issue in developmental biology. Reproduction. 2011; 142:377–81. 10.1530/REP-11-0118 21730109

[40] Bleil JD, Beall CF, Wassarman PM. Mammalian sperm-egg interaction: fertilization of mouse eggs triggers modification of the major zona pellucida glycoprotein, ZP2. Dev Biol. 1981; 86:189–97. 10.1016/0012-1606(81)90329-8 6793422

[41] Bleil JD, Wassarman PM. Sperm-egg interactions in the mouse: sequence of events and induction of the acrosome reaction by a zona pellucida glycoprotein. Dev Biol. 1983; 95:317–24. 10.1016/0012-1606(83)90032-5 6402397

[42] Nie J, Sui L, Zhang H, Zhang H, Yan K, Yang X, Lu S, Lu K, Liang X. Mogroside V protects porcine oocytes from *in vitro* ageing by reducing oxidative stress through SIRT1 upregulation. Aging (Albany NY). 2019; 11:8362–73. 10.18632/aging.102324 31586990 PMC6814602

[43] Kudryavtseva AV, Krasnov GS, Dmitriev AA, Alekseev BY, Kardymon OL, Sadritdinova AF, Fedorova MS, Pokrovsky AV, Melnikova NV, Kaprin AD, Moskalev AA, Snezhkina AV. Mitochondrial dysfunction and oxidative stress in aging and cancer. Oncotarget. 2016; 7:44879–905. 10.18632/oncotarget.9821 27270647 PMC5216692

[44] Aten RF, Duarte KM, Behrman HR. Regulation of ovarian antioxidant vitamins, reduced glutathione, and lipid peroxidation by luteinizing hormone and prostaglandin F2 alpha. Biol Reprod. 1992; 46:401–07. 10.1095/biolreprod46.3.401 1617013

[45] Sato EF, Kobuchi H, Edashige K, Takahashi M, Yoshioka T, Utsumi K, Inoue M. Dynamic aspects of ovarian superoxide dismutase isozymes during the ovulatory process in the rat. FEBS Lett. 1992; 303:121–25. 10.1016/0014-5793(92)80502-8 1607008

[46] Gasparrini B, Boccia L, Marchandise J, Di Palo R, George F, Donnay I, Zicarelli L. Enrichment of *in vitro* maturation medium for buffalo (bubalus bubalis) oocytes with thiol compounds: effects of cystine on glutathione synthesis and embryo development. Theriogenology. 2006; 65:275–87. 10.1016/j.theriogenology.2005.05.036 15979699

[47] Dickerhof N, Pearson JF, Hoskin TS, Berry LJ, Turner R, Sly PD, Kettle AJ, and AREST CF. Oxidative stress in early cystic fibrosis lung disease is exacerbated by airway glutathione deficiency. Free Radic Biol Med. 2017; 113:236–43. 10.1016/j.freeradbiomed.2017.09.028 28982600

[48] Lim J, Luderer U. Glutathione deficiency sensitizes cultured embryonic mouse ovaries to benzo[a]pyrene-induced germ cell apoptosis. Toxicol Appl Pharmacol. 2018; 352:38–45. 10.1016/j.taap.2018.05.024 29800640 PMC6013410

[49] Meister A. Mitochondrial changes associated with glutathione deficiency. Biochim Biophys Acta. 1995; 1271:35–42. 10.1016/0925-4439(95)00007-q 7599223

[50] Funahashi H, Cantley TC, Stumpf TT, Terlouw SL, Day BN. Use of low-salt culture medium for *in vitro* maturation of porcine oocytes is associated with elevated oocyte glutathione levels and enhanced male pronuclear formation after *in vitro* fertilization. Biol Reprod. 1994; 51:633–39. 10.1095/biolreprod51.4.633 7819443

[51] Sekhar RV, Patel SG, Guthikonda AP, Reid M, Balasubramanyam A, Taffet GE, Jahoor F. Deficient synthesis of glutathione underlies oxidative stress in aging and can be corrected by dietary cysteine and glycine supplementation. Am J Clin Nutr. 2011; 94:847–53. 10.3945/ajcn.110.003483 21795440 PMC3155927

[52] Chen D, Zhang XY, Sun J, Cong QJ, Chen WX, Ahsan HM, Gao J, Qian JJ. Asiatic acid protects dopaminergic neurons from neuroinflammation by suppressing mitochondrial ros production. Biomol Ther (Seoul). 2019; 27:442–49. 10.4062/biomolther.2018.188 30971058 PMC6720531

[53] Qi Z, Ci X, Huang J, Liu Q, Yu Q, Zhou J, Deng X. Asiatic acid enhances Nrf2 signaling to protect HepG2 cells from oxidative damage through Akt and ERK activation. Biomed Pharmacother. 2017; 88:252–59. 10.1016/j.biopha.2017.01.067 28110191

[54] Nataraj J, Manivasagam T, Justin Thenmozhi A, Essa MM. Neuroprotective effect of Asiatic acid on rotenone-induced mitochondrial dysfunction and oxidative stress-mediated apoptosis in differentiated SH-SYS5Y cells. Nutr Neurosci. 2017; 20:351–59. 10.1080/1028415X.2015.1135559 26856988

[55] Jiang W, Li M, He F, Bian Z, He Q, Wang X, Yao W, Zhu L. Neuroprotective effect of Asiatic acid against spinal cord injury in rats. Life Sci. 2016; 157:45–51. 10.1016/j.lfs.2016.05.004 27153777

[56] Dumollard R, Ward Z, Carroll J, Duchen MR. Regulation of redox metabolism in the mouse oocyte and embryo. Development. 2007; 134:455–65. 10.1242/dev.02744 17185319

[57] Yao X, Jiang H, Liang S, Shen X, Gao Q, Xu YN, Kim NH. Laminarin enhances the quality of aged pig oocytes by reducing oxidative stress. J Reprod Dev. 2018; 64:489–94. 10.1262/jrd.2018-031 30270255 PMC6305855

[58] Van Blerkom J. Mitochondria in human oogenesis and preimplantation embryogenesis: engines of metabolism, ionic regulation and developmental competence. Reproduction. 2004; 128:269–80. 10.1530/rep.1.00240 15333778

[59] May-Panloup P, Chretien MF, Malthiery Y, Reynier P. Mitochondrial DNA in the oocyte and the developing embryo. Curr Top Dev Biol. 2007; 77:51–83. 10.1016/S0070-2153(06)77003-X 17222700

[60] Ramalho-Santos J, Varum S, Amaral S, Mota PC, Sousa AP, Amaral A. Mitochondrial functionality in reproduction: from gonads and gametes to embryos and embryonic stem cells. Hum Reprod Update. 2009; 15:553–72. 10.1093/humupd/dmp016 19414527

[61] Michaels GS, Hauswirth WW, Laipis PJ. Mitochondrial DNA copy number in bovine oocytes and somatic cells. Dev Biol. 1982; 94:246–51. 10.1016/0012-1606(82)90088-4 6295849

[62] Smith LC, Alcivar AA. Cytoplasmic inheritance and its effects on development and performance. J Reprod Fertil Suppl. 1993; 48:31–43. 8145213

[63] Dumollard R, Duchen M, Carroll J. The role of mitochondrial function in the oocyte and embryo. Curr Top Dev Biol. 2007; 77:21–49. 10.1016/S0070-2153(06)77002-8 17222699

[64] Bisht S, Dada R. Oxidative stress: major executioner in disease pathology, role in sperm DNA damage and preventive strategies. Front Biosci (Schol Ed). 2017; 9:420–47. 10.2741/s495 28410127

[65] Roth Z. Symposium review: reduction in oocyte developmental competence by stress is associated with alterations in mitochondrial function. J Dairy Sci. 2018; 101:3642–54. 10.3168/jds.2017-13389 29395145

[66] Faitg J, Reynaud O, Leduc-Gaudet JP, Gouspillou G. [Skeletal muscle aging and mitochondrial dysfunction: an update]. Med Sci (Paris). 2017; 33:955–62. 10.1051/medsci/20173311012 29200393

[67] Zhang D, Keilty D, Zhang ZF, Chian RC. Mitochondria in oocyte aging: current understanding. Facts Views Vis Obgyn. 2017; 9:29–38. 28721182 PMC5506767

[68] Reynier P, May-Panloup P, Chrétien MF, Morgan CJ, Jean M, Savagner F, Barrière P, Malthièry Y. Mitochondrial DNA content affects the fertilizability of human oocytes. Mol Hum Reprod. 2001; 7:425–29. 10.1093/molehr/7.5.425 11331664

[69] Park JH, Seo YH, Jang JH, Jeong CH, Lee S, Park B. Asiatic acid attenuates methamphetamine-induced neuroinflammation and neurotoxicity through blocking of NF-kB/STAT3/ERK and mitochondria-mediated apoptosis pathway. J Neuroinflammation. 2017; 14:240. 10.1186/s12974-017-1009-0 29228978 PMC5725763

[70] Zhang X, Wu J, Dou Y, Xia B, Rong W, Rimbach G, Lou Y. Asiatic acid protects primary neurons against C2-ceramide-induced apoptosis. Eur J Pharmacol. 2012; 679:51–59. 10.1016/j.ejphar.2012.01.006 22296759

[71] Gao C, Wang F, Wang Z, Zhang J, Yang X. Asiatic acid inhibits lactate-induced cardiomyocyte apoptosis through the regulation of the lactate signaling cascade. Int J Mol Med. 2016; 38:1823–30. 10.3892/ijmm.2016.2783 27779647

[72] Elmore S. Apoptosis: a review of programmed cell death. Toxicol Pathol. 2007; 35:495–516. 10.1080/01926230701320337 17562483 PMC2117903

[73] Wang H, Jo YJ, Oh JS, Kim NH. Quercetin delays postovulatory aging of mouse oocytes by regulating SIRT expression and MPF activity. Oncotarget. 2017; 8:38631–41. 10.18632/oncotarget.16219 28418847 PMC5503559

[74] Isaev NK, Genrikhs EE, Oborina MV, Stelmashook EV. Accelerated aging and aging process in the brain. Rev Neurosci. 2018; 29:233–40. 10.1515/revneuro-2017-0051 29150992

